# Supplementary material for: How Many Sirtuin Genes Are Out There? Evolution of Sirtuin Genes in Vertebrates With a Description of a New Family Member
Source: Mol Biol Evol. 2023 Jan 20;40(2):msad014. doi: 10.1093/molbev/msad014 (PMC9897032; doi:10.1093/molbev/msad014)
Supplement: msad014_Supplementary_Data [file msad014_supplementary_data.zip › Supplementary_Figure1.pdf]

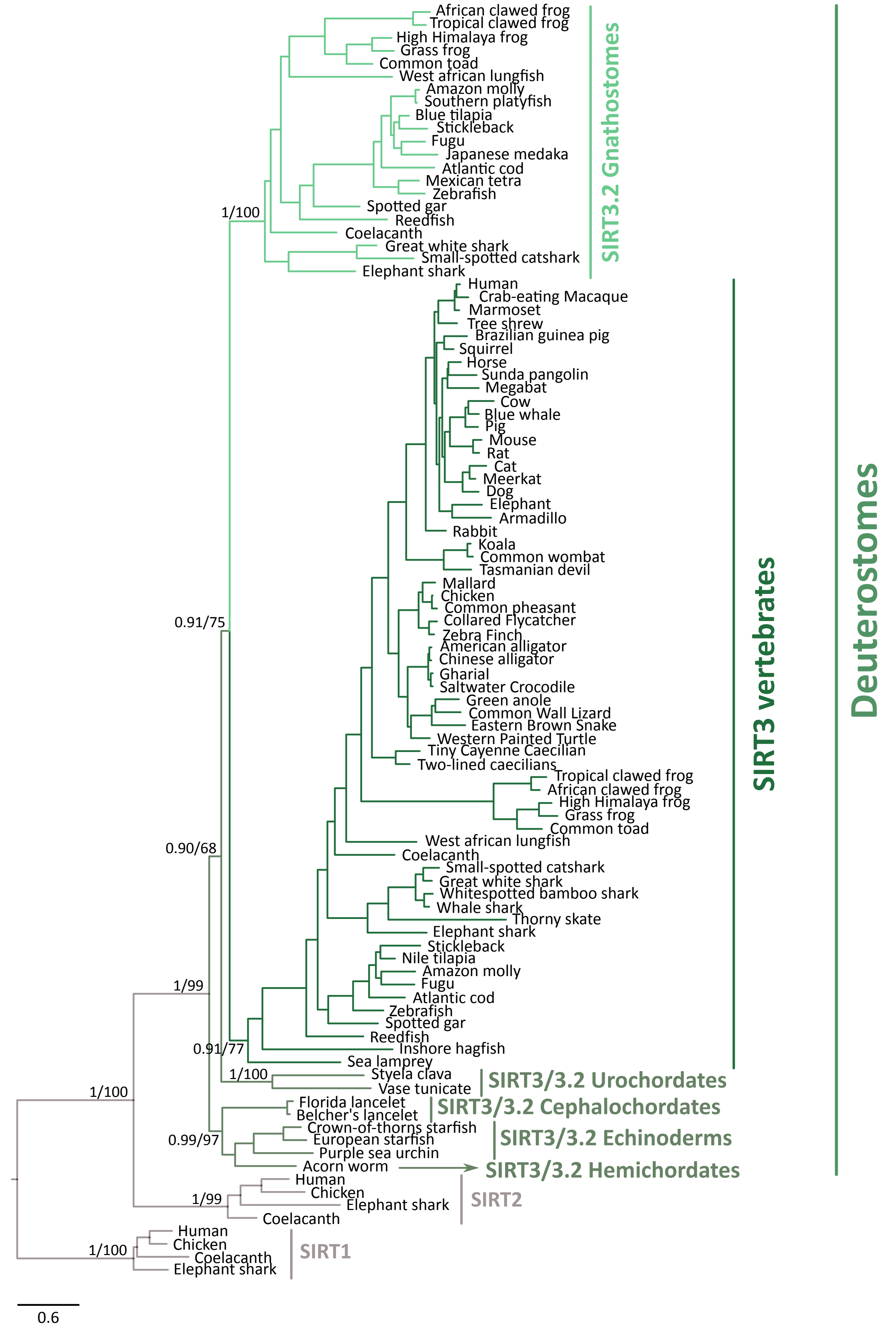

Supplementary figure 1. Maximum likelihood tree showing sister group relationships among SIRT3 and SIRT3.2 genes of deuterostomes. Numbers on the nodes correspond to support from the aBayes and ultrafast bootstrap values. SIRT1 and SIRT2 sequences from the human (*Homo sapiens*), chicken (*Gallus gallus*), coelacanth (*Latimeria chalumnae*) and elephant shark (*Callorhinchus milii*) were used as outgroups. The scale denotes substitutions per site and colors represent sirtuin lineages. Protein sequences were aligned using the software MAFFT v.7 (Katoh and Standley 2013), allowing the program to choose the alignment strategy (L-INS-i). To select the best-fitting model of molecular evolution we used the proposed model tool in the program IQ-Tree v1.6.12 (Kalyaanamoorthy et al. 2017), which selected JTT+I+G4. We used a maximum-likelihood approach to obtain the best tree using the program IQ-Tree v1.6.12 (Trifinopoulos et al. 2016). We carried out 20 independent runs to explore the tree space changing the value of the strength of the perturbation (-pers) parameter. We conducted the following analyses: five runs modifying the strength of the perturbation parameter from 0.5 (default value) to 0.3; five runs using the default value (0.5) for the strength of the perturbation parameter, five runs modifying the strength of the perturbation parameter from 0.5 (default value) to 0.7 and five runs modifying the strength of the perturbation parameter from 0.5 (default value) to 0.9. In all cases, the number of unsuccessful iterations to stop parameter (-nstop) value was changed from 100 (default value) to 500.
