# Supplementary material for: How Many Sirtuin Genes Are Out There? Evolution of Sirtuin Genes in Vertebrates With a Description of a New Family Member
Source: Mol Biol Evol. 2023 Jan 20;40(2):msad014. doi: 10.1093/molbev/msad014 (PMC9897032; doi:10.1093/molbev/msad014)
Supplement: msad014_Supplementary_Data [file msad014_supplementary_data.zip › Supplementary_Figure3.pdf]

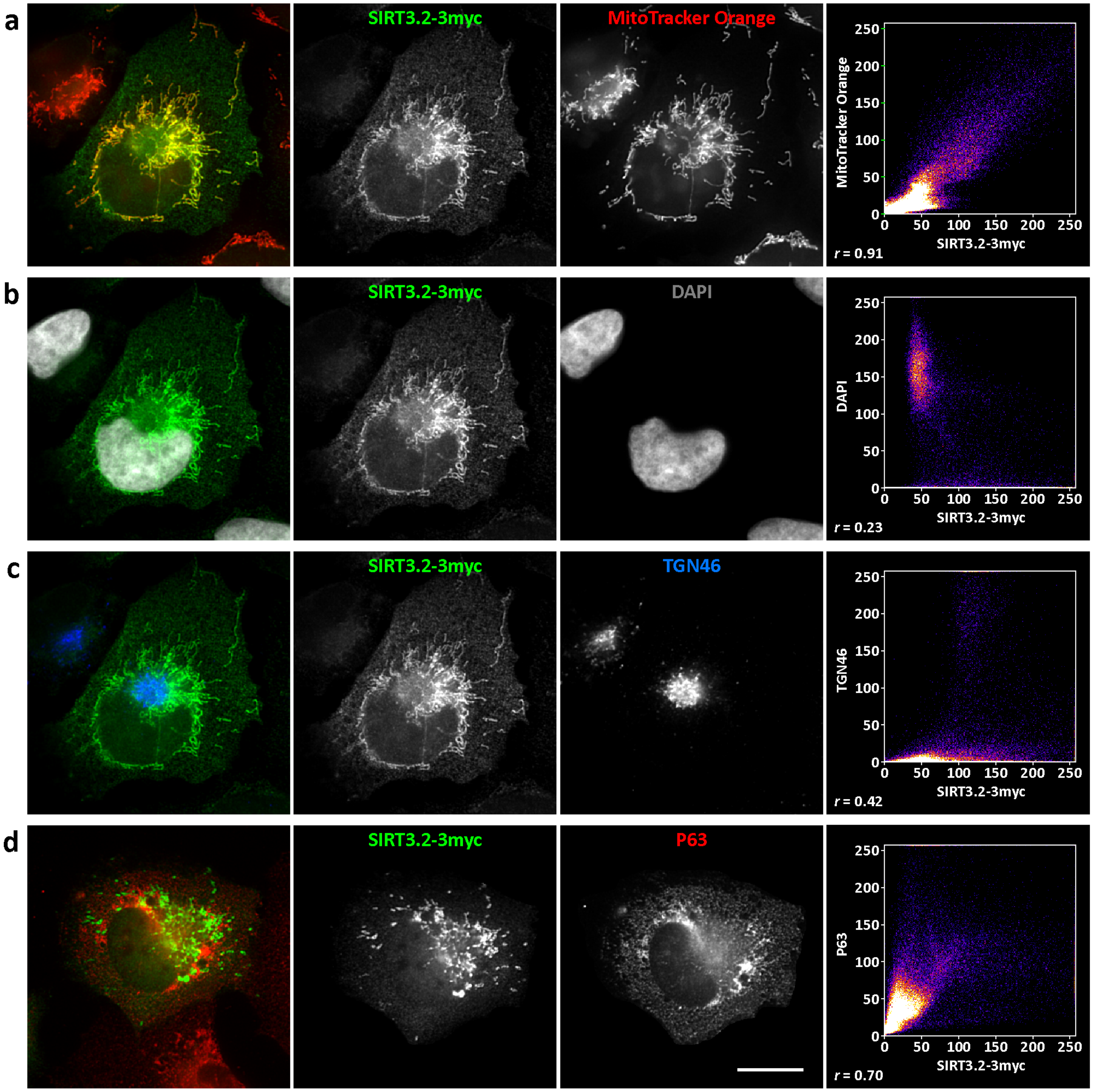

Supplementary Figure 3. Colocalization analysis of the immunofluorescence detection of SIRT3.2-3myc. H4 cells were processed as indicated in Figure legend 8c and 8d. The figure shows a representative colocalization analysis between the fluorescent signals of SIRT3.2-3myc and the mitochondrial probe MitoTracker Orange (a), SIRT3.2-3myc and the nuclear probe DAPI (b), SIRT3.2-3myc and antibody against the Golgi apparatus protein Trans-Golgi network integral membrane protein 2 (TGN46; c), and SIRT3.2-3myc and antibody against the endoplasmic reticulum protein Cytoskeleton-associated protein 4 (P63; d). The first column shows the overlapping of corresponding second and third images in each row. The fourth column shows the corresponding two-dimensional cytofluorograms of the colocalization analysis using the plugin JACoP implemented in the software ImageJ (version 1.47h). The corresponding Pearson's correlation coefficient is indicated in the bottom left corner of each cytofluorogram. Diagonal in a cytofluorogram indicates high degree of colocalization. Bar, 10 µm.
