# Supplementary material for: How Many Sirtuin Genes Are Out There? Evolution of Sirtuin Genes in Vertebrates With a Description of a New Family Member
Source: Mol Biol Evol. 2023 Jan 20;40(2):msad014. doi: 10.1093/molbev/msad014 (PMC9897032; doi:10.1093/molbev/msad014)
Supplement: msad014_Supplementary_Data [file Supplementary_Figure2.pdf › Supplementary_Figure2.pdf]

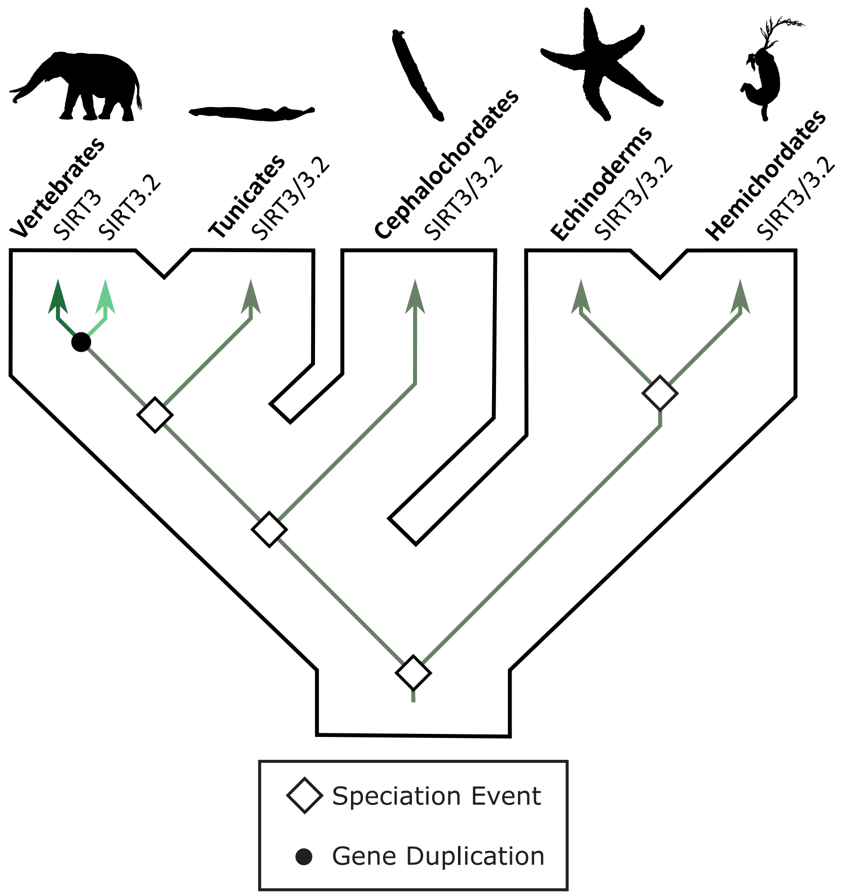

Supplementary figure 2. An evolutionary hypothesis regarding the evolution of the SIRT3 and SIRT3.2 genes in deuterostomes. According to our model, the last common ancestor of deuterostomes had a repertoire of one sirtuin gene (SIRT3/3.2), a condition maintained in hemichordates, echinoderms, cephalochordates, and tunicates. In the last common ancestor of vertebrates, the single gene copy underwent a duplication event giving rise to SIRT3 and SIRT3.2 genes. In the last common ancestor of amniotes, the group that includes mammals, birds, and reptiles, SIRT3.2 was lost (not shown). Silhouette images were obtained from PhyloPic (http://phylopic.org/).
